# Assessment of Gender Disparities in Short-Term and Long-Term Outcomes Following Posterior Fossa Tumor Resection

**DOI:** 10.7759/cureus.20000

**Published:** 2021-11-29

**Authors:** Ali S Farooqi, Starr Jiang, Austin J Borja, Donald K. E. D. Detchou, Ryan Dimentberg, Kaitlyn Shultz, Scott D McClintock, Neil R Malhotra

**Affiliations:** 1 Department of Neurosurgery, University of Pennsylvania Perelman School of Medicine, Philadelphia, USA; 2 Department of Mathematics, West Chester University, West Chester, USA

**Keywords:** social determinants of health, readmissions, gender, posterior fossa, brain tumor

## Abstract

Introduction

The analysis of social determinants of health (SDOH) across different surgical populations is critical for the identification of health disparities and the development risk mitigation strategies among vulnerable patients. Research into the impact of gender on neurosurgical outcomes remains limited. The aim of the present study was to assess the effect of gender on outcomes, in a matched sample, following posterior fossa tumor resection, a high-risk neurosurgical procedure.

Methods

Two hundred seventy-eight consecutive patients undergoing posterior fossa tumor resection over a six-year period (June 07, 2013, to April 29, 2019) at a single academic medical system were retrospectively evaluated. Short-term outcomes included 30- and 90-day rates of emergency department (ED) visit, readmission, reoperation, and mortality. Long-term outcomes included mortality and reoperation for the duration of follow-up. Firstly, male and female patients in the entire pre-match sample were compared. Thereafter, coarsened exact matching was employed to control for confounding variables, matching male and female patients on key demographic factors - including history of prior surgery, median household income, and race, amongst others - and outcome comparison was repeated.

Results

In both the entire pre-match sample and matched cohort analyses, no significant differences in adverse postsurgical events were discerned between the female and male patients when evaluating 30-day or 90-day rates of ED visit, readmission, reoperation, and mortality. There were also no differences in reoperation or mortality for the duration of follow-up.

Conclusion

Gender does not appear to impact short- or long-term outcomes following posterior fossa tumor resection. As such, risk assessment and mitigation strategies in this population should focus on other SDOH. Further studies should assess the role of other SDOH within this population.

## Introduction

The social determinants of health (SDOH) encompass patient characteristics that significantly impact health, such as gender, race, and level of education [1]. Early work has shown that interventions focused on SDOH have the potential to reduce morbidity, mortality, and total healthcare costs [2,3]. Recent policy changes, such as the Hospital Readmissions Reduction Program which ties Medicare reimbursement to readmission rates, have continued to move healthcare towards a value-based model of care and motivate reform to reduce readmissions, reoperations, and ED visits following treatment [4,5]. In response, hospitals have increasingly focused on SDOH as a way to reduce health inequity and total healthcare expenditure [1].

Gender is a particularly important SDOH, influencing social and biological factors, which may contribute to outcome disparities [6,7]. Socially, women face different societal expectations, stressors, and responsibilities with regards to their occupational and marital status, which impacts their social resources [8,9]. In terms of biological disparities, innate differences in genetics and lifetime hormonal changes may drive differences in health and susceptibility to disease between women and men [10,11].

In neurosurgery, gender-related outcome disparities have been reported following temporal lobectomy, vestibular schwannoma resection, glioblastoma resection, and traumatic brain injury (TBI) [12-15]. However, men and women were observed to have similar outcomes following other operations, including pituitary tumor resection, single-event multilevel surgery, and spinal nerve sheath tumor resection [16-18]. Due to the heterogeneous observations, further research is needed into the impact of gender on other neurosurgical procedures.

Posterior fossa tumor resections represent a high-risk neurosurgical patient population due to the sensitive nature of the surrounding anatomy [19,20]. Given the high rates of morbidity and surgical complications, the identification of SDOH that contribute to unfavorable outcomes following posterior fossa tumor resection is needed. Previous evidence in this population has confirmed that socioeconomic status contributes to adverse postsurgical events, but no studies yet have examined gender [21]. Here, we model the effects of gender on short-term and long-term outcomes across our broad population of posterior fossa tumor resection patients. Moreover, we employ coarsened exact matching (CEM) to control for confounding variables and isolate the impact of gender on outcomes.

## Materials and methods

Sample selection

**Figure 1:**
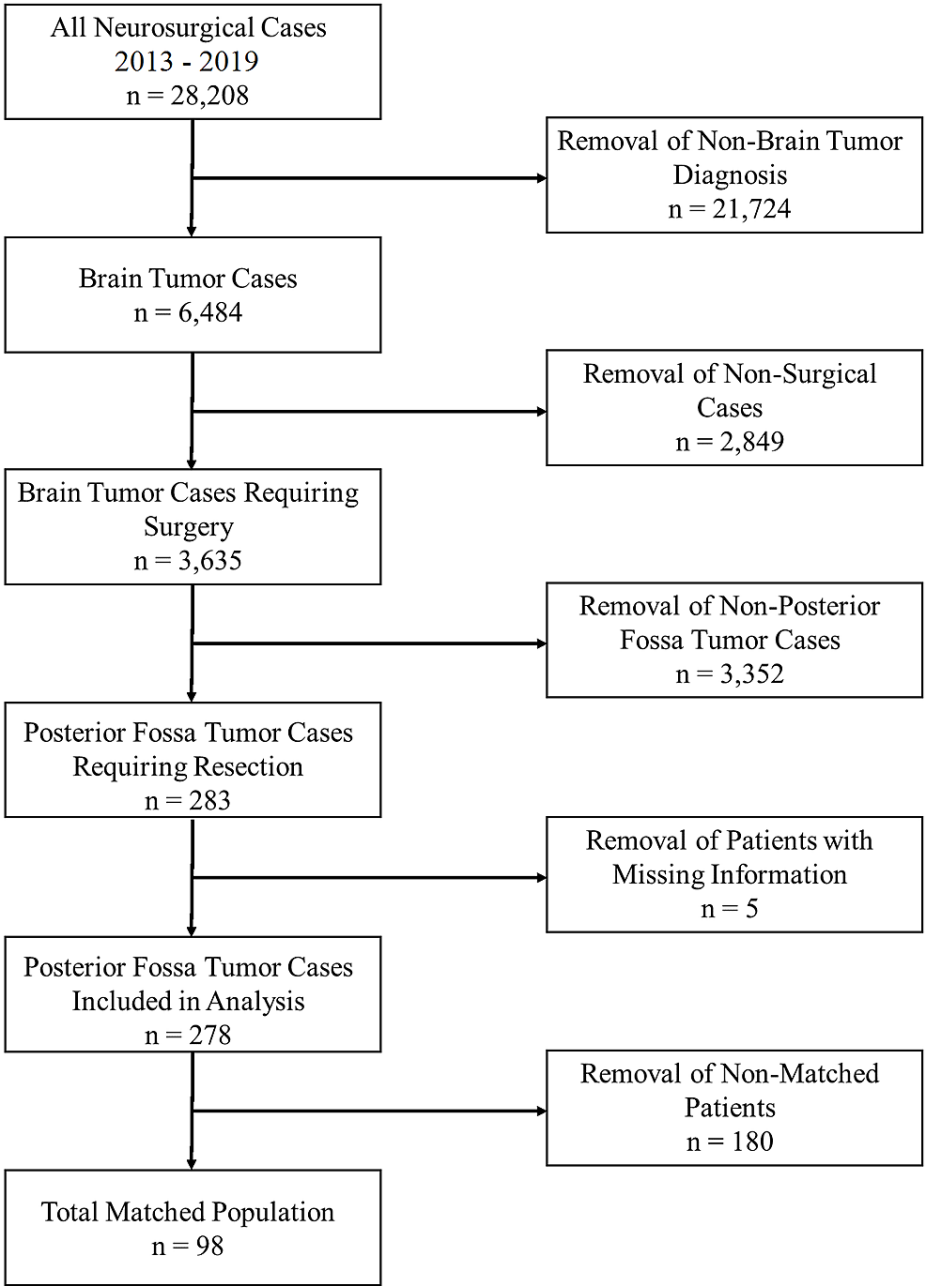
Patient selection. Flowchart describing selection of posterior fossa tumor cases from all neurosurgical procedure cases across a six-year period.

Two hundred eighty-three consecutive patients undergoing posterior fossa tumor resection at a multi-hospital, 1659-bed university health system over six years (June 07, 2013, to April 29, 2019) were retrospectively enrolled in this Institutional Review Board (IRB) approved study (Figure 1). Metastatic tumors were excluded (i.e., only primary posterior fossa tumors were included in this study). Patients with missing health information (n = 5) were excluded from the analysis, resulting in the pre-match sample (n = 278). The IRB considered this study to be minimal risk to patients and granted a waiver of informed consent. Key data were acquired using the EpiLog tool - a non-proprietary data acquisition system built and layered on top of the existing electronic health record architecture to facilitate charting, workflow, quality improvement, and cost reduction initiatives [22].

Data collection and matching

CEM was used to isolate and independently analyze the impact of gender on post-surgical outcomes. CEM generates 1:1 generates matches using the original matching variables [23]. An “exact match” is defined as a match on every covariate value. This contrasts with propensity score matching, which compacts the covariates into a single composite value for matching while ignoring the original covariates. CEM results in fewer patients included for analysis but offers superior control of confounders, and therefore is preferred to unmatched retrospective review.

Male and female patients were matched on multiple unique patient characteristics that have been associated with the degree of complexity and risk of adverse neurosurgical outcomes: median household income (MHI), race, level of education, insurance status, American Society of Anesthesiologists (ASA) grade, body mass index (BMI), Charlson Comorbidity Index (CCI) score and its underlying components, smoking status, prior resection of posterior fossa lesion vs primary resection, and any surgery in the 90 days prior to the index procedure. Binary matching was employed for MHI (above or below the median value for the dataset), insurance status (private or government insurance), and education level (percent without a high school diploma above or below the median value for the dataset). Ternary matching was employed for BMI, CCI, and ASA grade, with patients being binned into a low, medium, or high cohort. The remaining covariates were exactly matched (e.g., patients of African American race were matched 1:1). Unmatched patients were removed from the gender cohorts and not included in further analysis.

Statistical analysis

Short-term outcomes included ED visits, reoperation, readmission, and mortality within 30- and 90-days of the index operation. Long-term outcomes included reoperation and mortality, recorded for the duration of follow-up. These outcomes were evaluated because they are meaningful to patients, providers, and payers; they are utilized in all surgical subspecialties; their meanings are easily inferred; and their implications are readily applicable towards health policy. Each outcome measure was analyzed as a count, rather than categories with cutoffs. Patient characteristic and outcome data were extracted from EpiLog and transferred into defined spreadsheets.

All statistical analysis was performed via SAS Version 9.4 (SAS Institute Inc., Cary, North Carolina), except where specified otherwise. First, univariate analysis was performed for each outcome measure in the entire pre-match sample. Subsequently, for CEM, binning of the matching variables and removal of missing values was performed, and matching was completed using the MatchIt programming package in R Statistics (R Core Team, Vienna, Austria, 2017). McNemar’s test was used to compare the means between the two exact matched groups created via CEM. Finally, Bowker’s symmetry test, which aggregates all previously tested morbidity outcomes, was used to determine morbidity difference between groups across all metrics tested. Statistical significance for all analyses was set at a p-value < 0.05.

## Results

Patient characteristics

Within the pre-match sample (n = 278), female and male patients respectively had a mean age of 54.1 ± 14.4 and 55.5 ± 17.7, a BMI of 27.7 ± 7.1 and 27.0 ± 6.0, and a CCI score of 5.0 ± 3.6 and 5.5 ± 4.0, respectively (Table 1).

**Table 1:** Pre-match sample: patient characteristics. Demographic data describing the entire pre-match sample (n = 278) of patients undergoing posterior fossa tumor resection over six years.

Variable	Female (N = 150)	Male (N = 128)	Standardized difference
Age, mean (SD)	54.09 (14.40)	55.46 (17.70)	0.08
Median household income, USD, mean (SD)	74,730.53 (25,093.85)	69,956.98 (27,573.57)	-0.18
Race, n (%)			0.25
Asian	7 (4.67)	5 (3.91)	
Black/African American	23 (15.33)	18 (14.06)	
East Indian	1 (0.67)	2 (1.56)	
Hispanic/Latino	4 (2.67)	4 (3.12)	
Pacific Islander	1 (0.67)	0 (0)	
White	106 (70.67)	88 (68.75)	
Other	8 (5.33)	11 (8.60)	
Percentage with no diploma, mean (SD)	8.41 (5.42)	9.58 (5.95)	0.21
Insurance type n (%)			0.47
Medicare	32 (21.33)	48 (37.50)	
Medicaid	14 (9.33)	12 (9.38)	
Commercial	4 (2.67)	3 (2.34)	
Managed Care	77 (51.33)	59 (46.09)	
Self-pay	1 (0.67)	0 (0)	
Blue Cross	22 (14.67)	6 (4.69)	
American Society of Anesthesiologists Grade, n (%)			0.17
1	2 (1.33)	0 (0)	
2	37 (24.67)	33 (25.78)	
3	103 (68.67)	89 (69.53)	
4	8 (5.33)	6 (4.69)	
Body mass index, mean (SD)	27.71 (7.11)	26.99 (5.95)	-0.11
Charlson Comorbidity Index score, mean (SD)	5.00 (3.59)	5.54 (3.99)	0.14
Tobacco use within past 12 months, n (%)			0.27
Yes	18 (12.00)	14 (10.94)	
No	117 (78.00)	89 (69.53)	
Unknown	15 (10.00)	25 (19.53)	
Surgical interventions 90 days prior to the index operation, n (%)			0.20
0	137 (91.33)	113 (88.28)	
1	11 (7.33)	12 (9.38)	
2+	2 (1.33)	1 (0.78)	
Lifetime surgical interventions prior to the index operation, n (%)			0.18
0	133 (88.67)	114 (89.06)	
1	12 (8.67)	10 (7.81)	
2+	4 (2.47)	4 (3.13)	

Within the matched cohorts (n = 98), female and male patients, respectively, had a mean age of 54.5 ± 14.1 and 52.6 ± 17.6, a BMI of 27.0 ± 6.2 and 26.9 ± 5.2, and a CCI score of 4.6 ± 3.7 and 4.6 ± 3.7, respectively (Table 2).

**Table 2:** Matched cohorts: patient characteristics. Demographic data describing the coarsened exact matched cohorts (n = 98) of patients undergoing posterior fossa tumor resection over six years.

Variable	Female (N = 49)	Male (N = 49)	Standardized difference
Age, mean (SD)	54.53 (14.12)	52.55 (17.55)	-0.12
Median household income, USD, mean (SD)	77,040.12 (22,797.29)	77,239.45 (31,976.05)	0.01
Race, n (%)			0
Asian	2 (4.08)	2 (4.08)	
Black/African American	5 (10.20)	5 (10.20)	
Hispanic/Latino	1 (2.04)	1 (2.04)	
White	40 (81.63)	40 (81.63)	
Other	1 (2.04)	1 (2.04)	
Percentage with no diploma, mean (SD)	8.00 (4.91)	8.61 (6.53)	0.11
Insurance type n (%)			0.43
Medicare	13 (26.53)	13 (26.53)	
Medicaid	3 (6.12)	3 (6.12)	
Commercial	4 (8.16)	2 (4.08)	
Managed Care	22 (44.90)	29 (59.18)	
Blue Cross	7 (14.29)	2 (4.08)	
American Society of Anesthesiologists Grade, n (%)			0.07
1	0 (0)	0 (0)	
2	11 (22.45)	19 (38.78)	
3	38 (77.55)	30 (61.22)	
4	0 (0)	0 (0)	
Body mass index, mean (SD)	26.99 (6.23)	26.92 (5.24)	-0.01
Charlson Comorbidity Index score, mean (SD)	4.63 (3.72)	4.63 (3.71)	0
Tobacco use within past 12 months, n (%)			0
Yes	3 (6.12)	3 (6.12)	
No	41 (83.67)	41 (83.67)	
Unknown	5 (10.20)	5 (10.20)	
Surgical interventions 90 days prior to the index operation, n (%)			0
0	49 (100)	49 (100)	
1+	0 (0)	0 (0)	
Lifetime surgical interventions prior to the index operation, n (%)			0.29
0	48 (97.96)	48 (97.96)	
1	0 (0)	1 (2.04)	
2+	1 (2.04)	0 (0)	

Outcomes: pre-match sample

**Figure 2:**
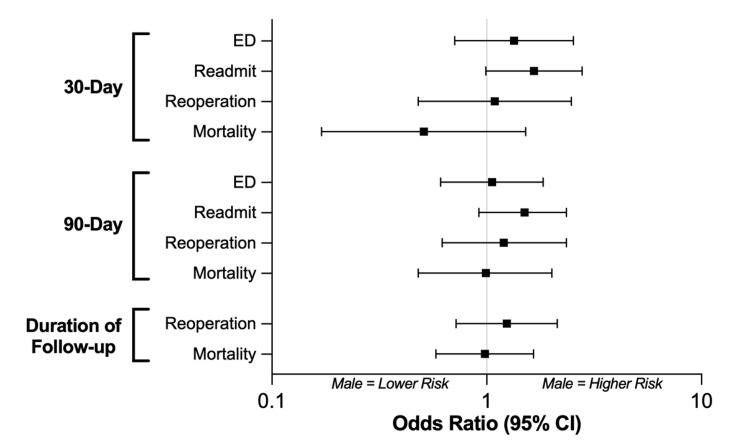
Pre-match sample: outcomes. Univariate logistic regression comparing outcome differences following posterior fossa tumor resection between male and female patients among the entire pre-match sample (n = 278). Odds ratios lower than 1 indicate that males have a lower risk of the outcome; odds ratios greater than 1 indicate that males have a higher risk. CI: 95% confidence interval, ED: emergency department.

Among the pre-match sample (n = 278), no significant differences were observed between female and male patients with respect to any short-term morbidity outcomes, including ED visits, readmissions, and reoperation within 30 or 90 days following the index operation (p = 0.06 - 0.85) (Figure 2, Table 3). Additionally, there was no difference in short-term mortality at either 30 days (p = 0.23) or 90 days (p = 0.97). Finally, no differences were observed in long-term outcomes, morality (p = 0.94) and reoperation (p = 0.44), over the entire duration of follow-up.

**Table 3:** Pre-match sample: outcomes. Patient outcome data among the entire pre-match sample (n = 278), as well as differences between male and female patients. ED: emergency department.

Outcome/complication	Female, n (%)	Male, n (%)	Odds ratio (95% confidence interval)	p-Value
30-Day ED Evaluation	22 (14.67)	24 (18.75)	1.34 (0.71 - 2.53)	0.36
30-Day Readmission	37 (24.67)	45 (35.16)	1.66 (0.99 - 2.78)	0.06
30-Day Reoperation	13 (8.67)	12 (9.38)	1.09 (0.48 - 2.48)	0.84
30-Day Mortality	11 (7.33)	5 (3.91)	0.51 (0.17 - 1.52)	0.23
90-Day ED Evaluation	36 (24.00)	32 (25.00)	1.06 (0.61 - 1.83)	0.85
90-Day Readmission	48 (32.00)	53 (41.41)	1.50 (0.92 - 2.45)	0.10
90-Day Reoperation	20 (13.33)	20 (15.63)	1.20 (0.62 - 2.35)	0.59
90-Day Mortality	19 (12.67)	16 (12.50)	0.99 (0.48 - 2.01)	0.97
Reoperation During Follow-up	35 (23.33)	35 (27.34)	1.24 (0.72 - 2.13)	0.44
Mortality During Follow-up	44 (29.33)	37 (28.91)	0.98 (0.58 - 1.65)	0.94

Outcomes: matched cohorts

**Figure 3:**
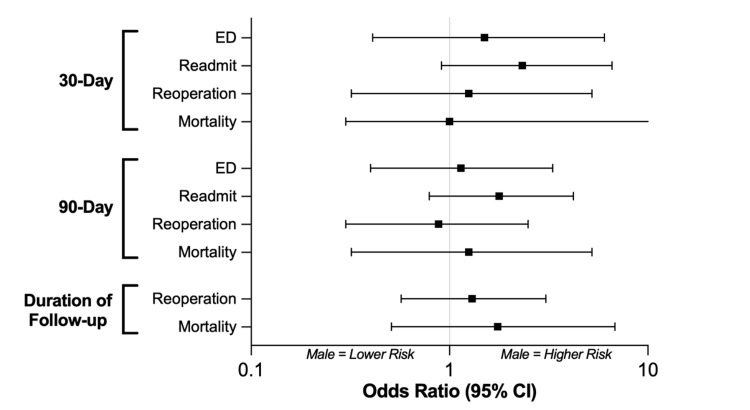
Matched cohorts: outcomes. Univariate logistic regression comparing outcome differences following posterior fossa tumor resection between male and female patients among the coarsened exact matched cohorts (n = 98). Odds ratios lower than 1 indicate that males have a lower risk of the outcome; odds ratios greater than 1 indicate that males have a higher risk. CI: 95% confidence interval, ED: emergency department.

Between the matched cohorts (n = 98), no significant differences were observed between female and male patients with respect to any short-term morbidity outcomes, including ED visits, readmissions, and reoperation within 30 or 90 days following the index operation (p = 0.08 - 0.80) (Figure 3, Table 4). Additionally, there was no difference in short-term mortality at either 30 days (p = 1.00) or 90 days (p = 0.75). Finally, no differences were observed in long-term outcomes, mortality (p = 0.39) and reoperation (p = 0.54), over the entire duration of follow-up.

**Table 4:** Post-match outcome data. Patient outcome data describing differences between male and female patients among the coarsened exact matched cohorts (n = 98). ED: emergency department.

Outcome/complication	Female, n (%)	Male, n (%)	Odds ratio (95% confidence interval)	p-value
30-Day ED Evaluation	4 (8.16)	6 (12.24)	1.50 (0.41 - 6.03)	0.55
30-Day Readmission	8 (16.33)	16 (32.65)	2.33 (0.91 - 6.60)	0.08
30-Day Reoperation	4 (8.16)	5 (10.20)	1.25 (0.32 - 5.23)	0.75
30-Day Mortality	1 (2.04)	1 (2.04)	1.00 (0.03 - 39.00)	1.00
90-Day ED Evaluation	10 (20.41)	11 (22.45)	1.14 (0.40 - 3.31)	0.80
90-Day Readmission	13 (26.53)	20 (40.82)	1.78 (0.79 - 4.21)	0.17
90-Day Reoperation	8 (16.33)	7 (14.29)	0.88 (0.30 - 2.49)	0.80
90-Day Mortality	5 (10.20)	6 (12.24)	1.25 (0.32 - 5.23)	0.75
Reoperation During Follow-up	13 (26.53)	16 (32.65)	1.30 (0.57 - 3.06)	0.54
Mortality During Follow-up	10 (20.41)	13 (26.53)	1.75 (0.51 - 6.82)	0.39

Bowker’s symmetry test demonstrated no significant difference between groups across a combined morbidity metric which aggregates all previously tested morbidity outcomes at both the 30-day (p = 0.16) and 90-day timepoints (p = 0.53). 

## Discussion

The present study evaluated the impact of gender among patients undergoing posterior fossa tumor resection. No significant differences were found in the pre-match or matched gender cohorts for any of the short-term mortality and morbidity measurements. Similarly, no significant differences were found in the pre-match or matched analyses for mortality and reoperation over the duration of follow-up.

This study contributes to the existing literature reporting the impact of SDOH on patient outcomes. Importantly, this study analyzed the effects of gender on posterior fossa tumor resection, a particularly high-risk operation due to the sensitivity of surrounding anatomical structures and high level of vascularity. Posterior fossa resection has a significant risk of morbidity and several complications, including new cranial nerve deficit, CSF leak, hematoma, and cerebellar edema [19,20]. Due to the high risk inherent to posterior fossa resections, complications tend to occur more frequently than their supratentorial counterparts, with an overall complication rate around 32% [24]. In this study, a similarly high rate of postoperative complications was seen, as reflected by 90-day ED visit and readmission rates of 24.5% and 36.3%, respectively.

Most studies have focused on the impact of SDOH on access to care. Low household income, low education level, and housing insecurity all contribute to economic and geographic barriers which make access to primary and specialty care especially difficult for patients of lower socioeconomic status [25]. Studies are limited, however, in terms of describing the effect of SDOH on patient outcomes once care is established. Initial research into income disparities among such neurosurgical populations provides useful initial guidance, but further investigation into other SDOH is necessary.

By characterizing the contribution of gender and SDOH to adverse patient outcomes across a broad population, departments and health systems can better risk-stratify patients and implement institution-wide interventions aimed at reducing morbidity for vulnerable patients. In the present study, gender was not observed to impact short- or long-term outcomes following posterior fossa tumor resection. Further studies should be employed to evaluate the impact of other SDOH on posterior fossa tumor resection outcomes. In addition, risk assessment and mitigation strategies in this broad population may be tailored toward other SDOH, such as income, that have been demonstrated to contribute to unfavorable outcomes.

Limitations

This retrospective study is inherently susceptible to data inaccuracies and sampling bias. Moreover, patient morbidity and mortality outcomes following resection were limited to events that are reported in our university-wide electronic medical record system, and therefore they may be underreported. However, the potential underreporting of patient outcomes is unlikely to affect the internal validity of the reported results, as it affects both female and male patients equally. Further, patients in the study sample received extensive follow-up (median follow-up of 703 days and 799 days for the female and male cohorts, respectively), during which patients were asked about all outside hospital encounters. Additional multicenter studies are warranted to confirm the present findings.

The primary outcomes evaluated herein were carefully selected because they are objective and widely utilized in surgical literature; they are meaningful to patients, providers, payers, and hospital systems; and they may be directly and immediately applicable to quality improvement efforts to support high-risk patients. Subjective metrics, such as quality of life, were not evaluated within this study. Future studies dedicated to studying gender disparities in posterior fossa tumor quality of life metrics are warranted.

The objective was to generate information regarding the broad impact of gender on outcomes broadly across this population of patients undergoing a high-risk surgical approach. It is possible that additional variables, not applied as matching covariates in the present study, may have served as confounders in analysis. This study did not report or analyze posterior fossa tumor characteristics, such as tumor pathology or size. However, this study was not intended to or adequately powered to assess differences in posterior fossa tumor pathology between male and female patients. Rather, the objective of this study was to evaluate gender in relation to adverse outcomes across a broad population of high-risk surgical patients. Further, the matching characteristics utilized herein were informed by the surgical literature, and each carefully selected covariate has previously been associated with postoperative outcomes. Numerous studies have demonstrated that race, smoking status, BMI, CCI score, and ASA grade each independently impact surgical morbidity and mortality [26-30]. Future, larger-scale studies are warranted to ascertain more granular information regarding the impact of gender following posterior fossa tumor resection.

## Conclusions

Gender does not predict adult patient outcomes following posterior fossa tumor resection. No significant differences were observed in short-term morbidity or mortality outcomes when comparing unmatched or matched patients following posterior fossa tumor resection. Similarly, no significant differences were found for mortality or reoperation for the duration of follow-up in either the unmatched or matched analyses. Future investigations should evaluate the impact of alternative SDOH on outcomes within this population. In addition, risk assessment and mitigation strategies, among patients requiring posterior fossa tumor resection, may potentially focus on other validated factors that contribute to unfavorable outcomes.
